# Methylsulfonylmethane: Applications and Safety of a Novel Dietary Supplement

**DOI:** 10.3390/nu9030290

**Published:** 2017-03-16

**Authors:** Matthew Butawan, Rodney L. Benjamin, Richard J. Bloomer

**Affiliations:** 1 Center for Nutraceutical and Dietary Supplement Research, School of Health Studies, The University of Memphis, Memphis, TN 38152, USA; mbbtawan@memphis.edu; 2 Bergstrom Nutrition, Vancouver, WA 98660, USA; Rbenjamin@bergstromnutrition.com

**Keywords:** methylsulfonylmethane, MSM, dimethyl sulfone, inflammation, joint pain

## Abstract

Methylsulfonylmethane (MSM) has become a popular dietary supplement used for a variety of purposes, including its most common use as an anti-inflammatory agent. It has been well-investigated in animal models, as well as in human clinical trials and experiments. A variety of health-specific outcome measures are improved with MSM supplementation, including inflammation, joint/muscle pain, oxidative stress, and antioxidant capacity. Initial evidence is available regarding the dose of MSM needed to provide benefit, although additional work is underway to determine the precise dose and time course of treatment needed to provide optimal benefits. As a Generally Recognized As Safe (GRAS) approved substance, MSM is well-tolerated by most individuals at dosages of up to four grams daily, with few known and mild side effects. This review provides an overview of MSM, with details regarding its common uses and applications as a dietary supplement, as well as its safety for consumption.

## 1. Description and History of MSM

Methylsulfonylmethane (MSM) is a naturally occurring organosulfur compound utilized as a complementary and alternative medicine (CAM) under a variety of names including dimethyl sulfone, methyl sulfone, sulfonylbismethane, organic sulfur, or crystalline dimethyl sulfoxide [1]. Prior to being used as a clinical application, MSM primarily served as a high-temperature, polar, aprotic, commercial solvent, as did its parent compound, dimethyl sulfoxide (DMSO) [2]. Throughout the mid-1950s to 1970s, DMSO was extensively studied for its unique biological properties including its membrane penetrability with and without the co-transport of other agents, its antioxidant capabilities, its anti-inflammatory effects, its anticholinesterase activity, and its ability to induce histamine release from mast cells [3]. After Williams and colleagues [4,5] studied the metabolism of DMSO in rabbits, others postulated that some of the biological effects attributed to DMSO may in part be caused by its metabolites [6].

In the late 1970s, Crown Zellerbach Corporation chemists, Dr. Robert Herschler and Dr. Stanley Jacob of the Oregon Health and Science University, began experimenting with the odorless MSM in search of similar therapeutic uses to DMSO [7]. In 1981 Dr. Herschler was granted a United States utility patent for the use of MSM to smooth and soften skin, to strengthen nails, or as a blood diluent [8]. In addition to the applications laid out in the first Herschler patent, subsequent Herschler patents claimed MSM to relieve stress, relieve pain, treat parasitic infections, increase energy, boost metabolism, enhance circulation, and improve wound healing [9,10,11,12,13,14,15,16], though there is little supporting scientific evidence [17]. On the other hand, the scientific literature does suggest that MSM may have clinical applications for arthritis [18,19,20] and other inflammatory disorders such as interstitial cystitis [21], allergic rhinitis [22,23], and acute exercise-induced inflammation [24].

Although MSM research has expanded since the patents of Herschler and one MSM product (OptiMSM^®^; Bergstrom Nutrition, Vancouver, WA, USA) was granted the Generally Recognized As Safe (GRAS) status by the Food and Drug Administration in 2007 [25], the use of MSM remained largely unchanged from 2002 to 2012 [26]. For example, according to the 1999–2004 National Health and Nutritional Examination Survey (NHANES), the weighted percentage of regular MSM users was 1.2% [27]. A 2007 study using a subjective survey reported that 9.6% of survey completers had tried MSM [28]; however, the sample of those who completed the survey was not diverse. More recent analysis of past data from the National Health Interview Surveys (NHIS) asserts that MSM use had dropped 0.2 percent points between 2007 and 2012 [26]. In more recent years, it appears that MSM use is on the rise, based on current MSM sales data.

### 1.1. MSM Synthesis—The Sulfur Cycle

MSM is a member of the methyl-*S*-methane compounds within the Earth’s sulfur cycle. Natural synthesis of MSM begins with the uptake of sulfate to produce dimethylsulfoniopropionate (DMSP) by algae, phytoplankton, and other marine microorganisms [29]. DMSP is either cleaved to form dimethyl sulfide (DMS) or undergoes demethiolation resulting in methanethiol, which can then be converted to DMS [30]. Approximately 1%–2% of the DMS produced in the oceans is aerosolized [29].

Atmospheric DMS is oxidized by ozone, UV irradiation, nitrate (NO_3_), or hydroxyl radical (OH) to form DMSO or sulfur dioxide [30,31,32,33,34,35]. Atmospheric levels of DMSO and MSM appear to be dependent upon the season with a maxima in the spring/summer and minima in the winter [36], possibly due to DMS production and volatility being temperature dependent. Oxidized DMS products like sulfur dioxide contribute to increased condensation and cloud formation [37,38], thus providing a vehicle for DMSO to return to Earth dissolved in precipitation where it can undergo disproportionation to either DMS or MSM [39].

Once absorbed into the soil, DMSO and MSM will be taken up by plants [40] or utilized by mutualistic soil bacterium such as the bioremediative additive, *Pseudomonas putida*, in order to improve soil conditions [41,42,43,44,45,46]. MSM is broadly expressed in a number of fruit [40,47], vegetable [40,47,48], and grain crops [47,49], though the extent of MSM bioaccumulation is dependent upon the plant. At this point, MSM and the other sulfur sources are consumed as a plant product and excreted, released as a by-product of plant respiration in the form of sulfide, or eventually decompose as the plant dies. The non-aerosolized sulfur sources can then be oxidized to sulfate and incorporated into minerals, which undergo erosion and return to the oceans, thus completing this sulfur sub-cycle.

Alternatively, synthetically produced MSM is manufactured through the oxidation of DMSO with hydrogen peroxide (H_2_O_2_) and purified by either crystallization or distillation. While distillation is more energy intensive, it is recognized as the preferred method [50] and utilized for manufacture of the GRAS OptiMSM^®^ (Bergstrom Nutrition, Vancouver, WA, USA) [25]. Biochemically, this manufactured MSM would have no detectable structural or safety differences from the naturally produced product [51]. Since the concentration of MSM is in the hundredths ppm in food sources, synthetically produced MSM makes it possible to ingest bioactive quantities without having to consume unrealistic amounts of food.

### 1.2. Absorption and Bioavailability

Exogenous sources of MSM are introduced into the body through supplementation or consumption of foods like fruits [40,47], vegetables [40,47,48], grains [47,49], beer [47], port wine [52], coffee [47], tea [47,53], and cow’s milk [47,54]. Along with MSM, absorbed methionine, methanethiol, DMS, and DMSO can be used by the microbiota to contribute to the MSM aggregate within the mammalian host [55,56,57]. Diet-induced microbiome changes have been shown to affect serum MSM levels in rats [58] and gestating sows [59]. That said, the gut flora is readily manipulated by diet [60], exercise [61], or other factors and likely affects bioavailable MSM sources, as suggested in pregnancy [62].

Pharmacokinetic studies indicate that MSM is rapidly absorbed in rats [63,64] and humans [65], taking 2.1 h and <1 h, respectively. Similar studies utilizing DMSO in monkeys demonstrate rapid conversion of DMSO to MSM within 1–2 h after delivery via oral gavage [66]. Humans ingesting DMSO oxidized approximately 15% to MSM by hepatic microsomes in the presence of NADPH_2_ and O_2_ [56].

In rats, between 59% and 79% of MSM is excreted the same day as administration in urine, either unchanged or as another *S*-containing metabolite [64]. Urine is the most common form of excretion as MSM has been detected in urine of rats [63,67], rabbits [4,5], bobcats [68], cheetahs [69], dogs [70], monkeys [66], and humans [4,62,71,72]. Additionally, excretion of MSM can be contained in feces [63,64] or several other biofluids including cow’s milk [54,73], red deer tail gland secretion [74], and human saliva [75].

The remaining MSM exhibits fairly homogeneous tissue distribution and a biological half-life of approximately 12.2 h in rats [63]. Tissue distribution in humans is also likely widespread as it has been detected in cerebrospinal fluid and evenly distributed between the gray and white matter of the brain [76,77,78,79,80]. Moreover, the biological half-life within the brain is an estimated 7.5 h [79], while the general half-life is suggested to be greater than 12 h [65]. The persisting systemic MSM comprises the bioavailable source.

MSM is a common metabolite with a steady state concentration dependent upon an assortment of individual-specific factors including, but not limited to, genetics [55,67,81] and diet [58,59,82]. In 1987 the first reported baseline MSM levels were 700–1100 ng/mL or 7.44–11.69 µmol/L [83]. Similar results have been observed with levels in the low micromolar range of 0–25 µmol/L [55]. More recently, a possible discrepancy has been noted in a study report listing baseline MSM levels ranging from 13.3 to 103 µM/mL [65]. In a recent human study involving daily ingestion of MSM at 3 g by 20 healthy men for a period of four weeks, it was noted that serum MSM was elevated in all men following ingestion, with a further increase at week 4 versus week 2 in the majority of men [84]. These data indicate that oral MSM is absorbed by healthy adults and accumulates over time with chronic intake.

## 2. Mechanisms of Actions

Due to its enhanced ability to penetrate membranes and permeate throughout the body, the full mechanistic function of MSM may involve a collection of cell types and is therefore difficult to elucidate. Results from in vitro and in vivo studies suggest that MSM operates at the crosstalk of inflammation and oxidative stress at the transcriptional and subcellular level. Due to the small size of this organosulfur compound, distinguishing between direct and indirect effects is problematic. In the sections to follow, an attempt will be made to describe each mechanism within a focused scope.

### 2.1. Anti-Inflammation

In vitro studies indicate that MSM inhibits transcriptional activity of nuclear factor kappa-light-chain-enhancer of activated B cells (NF-κB) [85,86] by impeding the translocation into the nucleus while also preventing the degradation of the NF-κB inhibitor [86]. MSM has been shown to alter post-translational modifications including blocking the phosphorylation of the p65 subunit at Serine-536 [87], though it is unclear whether this is a direct or indirect effect. Modifications to subunits such as these contribute heavily to the regulation of the transcriptional activity of NF-κB [88], and thus more details are required to further understand this anti-inflammatory mechanism. Traditionally, the NF-κB pathway is thought of as a pro-inflammatory signaling pathway responsible for the upregulation of genes encoding cytokines, chemokines, and adhesion molecules [89]. The inhibitory effect of MSM on NF-κB results in the downregulation of mRNA for interleukin (IL)-1, IL-6, and tumor necrosis factor-α (TNF-α) in vitro [90,91]. As expected, translational expression of these cytokines is also reduced; furthermore, IL-1 and TNF-α are inhibited in a dose-dependent manner [90].

MSM can also diminish the expression of inducible nitric oxide synthase (iNOS) and cyclooxygenase-2 (COX-2) through suppression of NF-κB; thus lessening the production of vasodilating agents such as nitric oxide (NO) and prostanoids [86]. NO not only modulates vascular tone [92] but also regulates mast cell activation [93]; therefore, MSM may indirectly have an inhibitory role on mast cell mediation of inflammation. With the reduction in cytokines and vasodilating agents, flux and recruitment of immune cells to sites of local inflammation are inhibited.

At the subcellular level, the nucleotide-binding domain, leucine-rich repeat family pyrin domain containing 3 (NLRP3) inflammasome senses cellular stress signals and responds by aiding in the maturation of inflammatory markers [94,95]. MSM negatively affects the expression of the NLRP3 inflammasome by downregulating the NF-κB production of the NLRP3 inflammasome transcript and/or by blocking the activation signal in the form of mitochondrial generated reactive oxygen species (ROS) [90]. The mechanisms by which MSM demonstrates antioxidant properties will be discussed in the following section.

### 2.2. Antioxidant/Free-Radical Scavenging

Although an excess of ROS can wreak havoc on a number of intracellular components, a threshold amount is required to activate the appropriate pathways in phenotypically normal cells [96]. The antioxidant effect of MSM was first noticed when the neutrophil stimulated production of ROS was suppressed in vitro but unaffected in a cell free system [97]; for that reason, it was proposed that the antioxidant mechanism acts on the mitochondria rather than at the chemical level.

MSM influences the activation of at least four types of transcription factors: NF-κB, signal transducers and activators of transcription (STAT), p53, and nuclear factor (erythroid-derived 2)-like 2 (Nrf2). By mediating these transcription factors, MSM can regulate the balance of ROS and antioxidant enzymes. It is important to note that each of these is also, in part, activated by ROS. 

As mentioned previously, MSM can inhibit NF-κB transcriptional activity and thus reduce the expression of enzymes and cytokines involved in ROS production. Downregulation of COX-2 and iNOS reduces the amount of superoxide radical (O_2_^−^) and nitric oxide (NO), respectively [86]. Additionally, MSM suppresses the expression of cytokines such as TNF-α [86,90,91], which may reduce any stimulated mitochondrial generated ROS [98]. Decrements in cytokine expression may also be involved in reduced paracrine signaling and activation of other transcription factors and pathways.

MSM has been shown to repress the expression or activities of STAT transcription factors in a number of cancer cell lines in vitro [99,100,101]. The janus kinase (Jak)/STAT pathway is involved in regulation of genes related to apoptosis, differentiation, and proliferation, all of which generate ROS as a necessary signaling component [102,103,104]. Signaling through the Jak/STAT pathway may also be stifled by reduced cytokine expression. Downregulation of the Jak/STAT pathway may further reduce ROS generation by decreasing expression of oxidases [105] and B-cell lymphoma-2 (Bcl-2) [106].

In macrophage-like cells, pre-treatment with MSM in vitro was found to decrease accumulation of the redox sensitive p53 transcription factor [107]. This p53 exhibits dichotomous oxidative function depending on the intracellular ROS levels, whereby, in a general sense, p53 exerts antioxidative functions at low intracellular ROS levels and prooxidative functions at high ROS levels [108]. The antioxidative function of p53 upregulates scavenging enzymes like Sestrin, glutathione peroxidase (GPx) and aldehyde dehydrogenase (ALDH). The prooxidative function of p53 upregulates oxidases while also suppressing antioxidant genes. For a more in depth summary of p53 and oxidative stress, please see the review by Liu and Xu [108].

Murine neuroblastoma cells cultured with human immunodeficiency virus type 1 transactivating regulatory protein (HIV-1 Tat) displayed reduced nuclear translocation of Nrf2; however, co-culturing with MSM returned Nrf2 translocation to the nucleus to control levels [109]. Nrf2 is well documented for its association with antioxidant enzymes including glutamate-cysteine ligase (GCL), superoxide dismutases (SODs), catalase (CAT), peroxiredoxin (Prdx), GPx, glutathione S-transferase (GST), and others [110]. Though it is unclear what direct effect MSM has on Nrf2, it is worth mentioning that Nrf2 can also be regulated by p53 expression of p21 or Jak/STAT expression of B-cell lymphoma-extra large (Bcl-XL) [111].

### 2.3. Immune Modulation

Stress can trigger an acute response by the innate immune system and an ensuing adaptive immune response if the stressor is pathogenic. Sulfur containing compounds including MSM play a critical role in supporting the immune response [112,113,114]. Through an integrated mechanism including those mentioned above, MSM modulates the immune response through the crosstalk between oxidative stress and inflammation.

Chronic exposure to stressors can have detrimental effects to the immune system as it becomes desensitized or over-stressed and unable to elicit a typical immune response. The broad effects of IL-6 have been implicated in the maintenance of chronic inflammation [115]. MSM has been shown to reduce IL-6 in vitro, which may mitigate these chronic deleterious effects [86,87,90]. Pre-treatment with MSM, prior to exhaustive exercise, prevented the over-stress of immune cells as lipopolysaccharide (LPS)-treated blood was still able to mount a response through the secretion of cytokines ex vivo, an effect not observed in the placebo group [24].

The adjacent vasculature plays a role in mediating the acute immune response primarily through the activation of mast cells. Histamine release from mast cells is inhibited by DMSO [116]; however, the effects of MSM on histamine release remain unexplored. Previous studies indicate that MSM has an inhibitory role on vascular function [117,118]. Other in vitro studies demonstrate that MSM has the ability to dampen the expression of vasodilating agents such as NO and prostanoids [86]. A reduction in NO protects macrophages against NO stimulated apoptosis [107].

Additionally, MSM may serve other immune modulatory effects related to cell cycle and cell death. In vitro studies indicate that MSM can induce apoptosis in gastrointestinal cancer cells [119], hepatic cancer cells [120], and colon cancer cells [121]. Contrary to these findings, MSM did not induce apoptosis in murine breast cancer cells [122]. Rather, MSM was shown to restore normal cellular metabolism to both metastatic murine breast cancer and murine melanoma cells [123]. Cell cycle arrest has also been observed in gastrointestinal cancer cells [119] and myoblasts [124]. These alterations to cell survival may arise from cyclin production modulations to the p53 and Jak/STAT pathways.

Though few studies have examined the effectiveness of MSM on wound healing, the innate immune system may also benefit from enhanced wound closure, as assessed by the scratch test in vitro [124,125,126]. Future studies would be needed to confirm these results in vivo.

### 2.4. Sulfur Donor/Methylation

MSM has long been thought of as a sulfur donor for sulfur containing compounds such as methionine, cysteine, homocysteine, taurine, and many others. Guinea pigs fed radiolabeled MSM incorporated labeled sulfur into serum proteins containing methionine and cysteine [127]. This study suggested that microbial metabolism may be responsible for the utilization of MSM to form methionine and subsequent synthesis to cysteine. More recent in vivo studies with radiolabeled MSM suggest that this compound is metabolized rapidly in a homogenous distribution of tissues [63,64]. These studies reportedly collected most labeled sulfur as metabolites of MSM in urine but did not determine the metabolites. Further study regarding the activity of MSM as a sulfur donor is ongoing.

In humans, no MSM dose-dependent trends are observed between individuals for plasma sulfate and homocysteine changes [65]. With microorganisms largely responsible for sulfur utilization throughout the sulfur cycle, MSM as a sulfur donor may be dependent on the existing microbiome with mammalian hosts.

MSM is reportedly a non-alkylating agent and does not methylate DNA [128]. In a letter by Kawai et al., the parent compound of MSM, DMSO, can methylate DNA in the presence of hydroxyl radical (OH) [129], which also has the potential to aid in the oxidation of DMSO to MSM [32,35]. Although it is uncertain whether MSM alkylates DNA, MSM does not appear to cause chromosome aberration in vitro or micronucleation in vivo according to two final study reports. Future studies are needed to determine whether MSM is a methyl donor.

## 3. Common Uses

As a therapeutic agent, MSM utilizes its unique penetrability properties to alter physiological effects at the cellular and tissue levels. Furthermore, MSM has the ability to act as a carrier or co-transporter for other therapeutic agents, even furthering its potential applications.

### 3.1. Arthritis and Inflammation

Arthritis is an inflammatory condition of the joints that currently affects approximately 58 million adults, with an estimated increase to 78.4 million by 2040 [130]. This inflammation is characterized by pain, stiffness, and a reduced range of motion with regards to the arthritic joint(s). MSM is currently a CAM treatment alone and in combination for arthritis and other inflammatory conditions. MSM, as a micronutrient with enhanced penetrability properties, is commonly integrated with other anti-arthritic agents including glucosamine, chondroitin sulfate, and boswellic acid.

As mentioned previously, a number of in vitro studies suggest that MSM exerts an anti-inflammatory effect through the reduction in cytokine expression [86,87,90,91]. Similar results have been observed with MSM in experimentally induced-arthritic animal models, as evidenced by cytokine reductions in mice [131] and rabbits [86,87,90,91,132]. Additionally, MSM in a combinatorial supplement with glucosamine and chondroitin sulfate effectively reduced C-reactive protein (CRP) in rats with experimentally-induced acute and chronic rheumatoid arthritis [133].

To date, most arthritic human studies have been non-invasive and assess joint condition through the use of subject questionnaires such as the Western Ontario and McMaster Universities Arthritis Index (WOMAC), 36-Item Short Form Survey (SF36), Visual Analogue Scale (VAS) pain, and the Lequesne Index. In his overview of MSM, Dr. Stanley Jacob references eleven case studies of patients suffering from osteoarthritis who experienced improved symptoms following supplementation with MSM [7]. Clinical trials suggest MSM is effective in reducing pain, as indicated by the VAS pain scale [18,134], WOMAC pain subscale [18,19,135,136], SF36 pain subscale [18,136], and Lequesne Index [134]. Concurrent improvements were also noted in stiffness [18,135,136] and swelling [134]. Furthermore, in the study conducted by Usha and Naidu [134], MSM in combination with glucosamine potentiated the improvements in pain, pain intensity, and swelling.

Other human studies utilizing combination therapies report similar results. For instance, arthritis associated pain and stiffness was significantly improved through the use of Glucosamine, Chondroitin sulfate, and MSM (GCM) [137,138]. Only marginal improvements in pain and stiffness were observed when a GCM combination was supplemented on top of modifications to diet and exercise in sedentary obese women diagnosed with osteoarthritis (OA) [139]. MSM was also shown to be effective in reducing arthritis pain when used in combination with boswellic acid [140] and type II collagen [141].

In addition to arthritis, MSM improves inflammation in a number of other conditions. For example, MSM attenuated cytokine expression in vivo for induced colitis [142], lung injury [143], and liver injury [143,144]. Hasegawa and colleagues [131] reported that MSM was useful in protecting against UV-induced inflammation when applied topically and acute allergic inflammation after pre-treatment with a 2.5% aqueous drinking solution.

MSM is effective at reducing other inflammatory pathologies in humans as well. In a physician’s review of clinical case studies, MSM was an effective treatment for four out of six patients suffering from interstitial cystitis [21]. Additionally, MSM is also suggested to alleviate the symptoms of seasonal allergic rhinitis [22,23]. Though the reduction in systemic exercise-induced inflammation by MSM has been observed [24], human studies have not explored the inflammatory effects directly at the cartilage or synovium, as seen in the reduced synovitis inflammation in mice given MSM [145].

### 3.2. Cartilage Preservation

Cartilage degradation has long been thought of as the driving force of osteoarthritis [146]. Articular cartilage is characterized by a dense extracellular matrix (ECM) with little to no blood supply driving nutrient extraction from the adjacent synovial fluid [147]. Pro-inflammatory cytokines, particularly IL-1β and TNF-α, are implicated in the destructive process of cartilage ECM [148]. With minimal blood supply and possible hypoxic microenvironments, in vitro studies suggest that MSM protects cartilage through its suppressive effects on IL-1β and TNF-α [86,90,91] and its possibly normalizing hypoxia-driven alterations to cellular metabolism [123]. 

Disruption of this destructive autocrine or paracrine signaling by MSM has also been observed in surgically-induced OA rabbits by the reduction in cartilage and synovial tissue [132], TNF-α, and the protected articular cartilage surface during OA progression. Histopathology of a rheumatoid arthritis (RA) rat model supplemented with a GCM combination demonstrated decreased synovium proliferation and the development of an irregular edge at the articular joint [133]. Furthermore, MSM supplementation in OA mice significantly decreased cartilage surface degeneration [149]. In fact the protective effects of MSM can be seen as far back as 1991, when Murav’ev and colleagues described the decreased knee joint degeneration of arthritic mice [150]. Interestingly, endogenous serum MSM becomes elevated in sheep post-meniscal destabilization caused osteoarthritis [151]; however, the magnitude of this physiological response was not large enough to protect against cartilage erosion.

### 3.3. Improve Range of Motion and Physical Function

With the aforementioned improvements in inflammation and cartilage preservation, not surprisingly beneficial changes in overall physical function have also been noted through the use of subjective measurements [18,19,135,136]. In studies with osteoarthritic populations given MSM daily, significant improvements in physical function were observed, as assessed through the WOMAC [18,19,135,136], SF36 [19,135,136], and Aggregated Locomotor Function (ALF) [135]. Objective kinetic knee measurements following eccentric exercise-induced muscle damage were not conclusive but suggest that MSM may aid in maximal isometric knee extensor recovery [152].

MSM has been used in a number of combination therapies with positive results. Supplementation with glucosamine, chondroitin sulfate, MSM, guava leaf extract, and Vitamin D improved physical function in patients with knee osteoarthritis based on the Japanese Knee OA Measure [137]. A GCM supplement was successful in increasing functional ability and joint mobility [138]. MSM in combination with boswellic acid was also shown to improve knee joint function as assessed through the Lequesne Index [140]. MSM with arginine l-α-ketoglutarate, hydrolyzed Type I collagen, and bromelain taken for three months daily post-rotator cuff repair improved repair integrity without affecting objective functional outcomes [153].

Other studies exploring the uses of MSM in combination therapies failed to show significant improvements. In one such study in geriatric horses, a GCM combination supplement given orally for three months failed to show significant changes in gait characteristics [154]. In humans, MSM and boswellic acid reduced the need for anti-inflammatory drugs but was not more effective than the placebo as a treatment for gonarthrosis [155]. However, when a GCM combination supplement was administered in addition to dietary and exercise interventions, no significant improvements were noted when compared to the non-supplemented group [139].

Subjects with lower back pain undergoing conventional physical therapy with supplementation of a glucosamine complex containing MSM reported an improvement in their quality of life [156]. A 2011 systematic review of GCM supplements as a treatment for spinal degenerative joint disease and degenerative disc disease failed to come to a conclusion on efficacy due to the scarcity of quality literature [157].

### 3.4. To Reduce Muscle Soreness Associated with Exercise

Prolonged strenuous exercise can result in muscle soreness caused by microtrauma to muscles and surrounding connective tissue leading to a local inflammatory response [158]. MSM is alluded to be an effective agent against muscle soreness because of its anti-inflammatory effects as well as its possible sulfur contribution to connective tissue. Endurance exercise-induced muscle damage was reduced with MSM supplementation, as measured by creatine kinase [159]. Pre-treatment with MSM reduced muscle soreness following strenuous resistance exercises [152,160,161] and endurance exercise [162].

### 3.5. Reduce Oxidative Stress

In vitro studies suggest that MSM does not chemically neutralize ROS in stimulated neutrophils but instead suppresses mitochondrial generation of superoxide, hydrogen peroxide, and hypochlorous acid [97]. Additionally, MSM is able to restore the reduced glutathione (GSH)/oxidized glutathione (GSSG) ratio to normal levels, decrease NO production, and reduce neuronal ROS production following HIV-1 Tat exposure [109]. Animal studies using MSM as the primary treatment for experimentally induced injuries show reductions in malondialdehyde (MDA) [142,143,144,163,164,165], GSSG [165], myeloperoxidase (MPO) [142,143,163], NO [164], and carbon monoxide (CO) [164] and/or increases in GSH [142,143,163,164,165,166], CAT [142,143,144,165], SOD [143,144,163,165], and GPx [165]. Treatment modalities for these animal studies were either an acute one time dose or pre-treatment prior to inducing injury [144,163,165].

In humans, MSM pre-treatment prior to endurance exercise results in acute attenuation of induced protein oxidation [167,168], bilirubin [159,168], lipid peroxidation [167], creatine kinase [159], oxidized glutathione [167], and uric acid [168] and also an increase in total antioxidant capacity [159,168]. Following endurance exercise, reduced glutathione was elevated with 10 days of pre-treatment [167] but was insignificantly affected by a single oral dose just prior to exercise [168].

Pre-treatment with MSM in subjects undergoing resistance exercise exhibits more variability. Supplementation for 28 days with 3.0 g/day prior to exhaustive resistance exercise showed an increase in Trolox equivalent antioxidant capacity (TEAC) and a decrease in homocysteine [161]; whereas, supplementation for 14 days at the same dosage reported no significant changes in TEAC or homocysteine [160]. The longer period of supplementation may have allowed bioavailable MSM stores to reach a level where it could upregulate Nrf2 enough to produce a more significant rise in antioxidant enzymes.

Combination therapies including MSM have become more popular recently, particularly with ethylenediaminetetraacetic acid (EDTA) due to the permeability enhancement provided by MSM [169]. For instance, topical EDTA-MSM is effective at reducing oxidative damage in the form of protein-lipid aldehyde adducts [170,171,172]. EDTA-MSM reduced lens opacification in diabetic cataract [172] but was ineffective in reversing experimentally induced intraocular pressure in rats [170]. In humans, EDTA-MSM lotion significantly improved pitting edema symptoms after two weeks of application, with circulating total antioxidant capacity and MDA reductions noted [173].

Humans studies show promise for MSM as an antioxidant with similar results noted, including reductions in MDA [19,167,168], protein carbonyls (PC) [167,168], and uric acid [168] and increases in GSH [167] and TEAC [159,161,168]. Contrary to previous literature, Kantor et al. reported that MSM users experienced reduced lymphocyte DNA repair capacity at 60 min. [174]. This conflicting result may be explained by the samples being collected at different points in the day, since the circadian clock can modulate this measure [175].

### 3.6. Improve Seasonal Allergies

In an evaluation of MSM on seasonal allergies, 2.6 g/day PO MSM for 30 days improved upper and total respiratory symptoms as well as lower respiratory symptoms by week 3 [23]. All these improvements were maintained throughout the 30 days of supplementation. A drawback of this study was the lack of reporting on pollen counts and a symptoms questionnaire [176]. This was later corrected when Barrager and Schauss published the additional requested data [22]. Barrager et al. used a subsection of this sample population to measure histamine release but found no significant changes in plasma IgE or histamine levels [23].

### 3.7. Improve Skin Quality and Texture

Since the initial patent awarded to Herschler in 1981, MSM has been suggested to have therapeutic uses for the improvement of skin quality and texture by acting as a sulfur donor to keratin. According to one final study report, MSM is non-irritating to the skin of rabbits via an occlusive patch. Another final study report indicated that MSM may be slightly irritating to skin of guinea pigs. Using a lotion containing EDTA and MSM, mild improvement in burn sites on rats were noticed following three days of topical application every 8 h [171].

Skin appearance and condition after MSM treatment significantly improved as assessed by expert grading, instrumental analysis, and participant self-assessment [177]. Human combination studies with four peeling sessions using pyruvic acid and MSM once every two weeks improved the degree of pigmentation of melisma, skin elasticity, and the degree of wrinkling [178]. A combination treatment of silymarin and MSM proved useful in managing rosacea symptoms [179]. A case study of a 44 year old man with severe X-linked type ichthyosis showed improvement of symptoms after four weeks of topical moisturizer containing amino acids, vitamins, antioxidants, and MSM [180].

### 3.8. MSM and Cancer

An emerging area of MSM research deals with the anti-cancer effect of the organosulfur compound. In vitro studies using MSM alone or in combination have evaluated the metabolic and phenotypic effects of a number of cancer cell lines including breast [100,101,122,123,126,181], esophagus [119], stomach [119], liver [119,120], colon [121], bladder [99], and skin cancers [123,125] with promising results. MSM independently has been shown to be cytotoxic to cancer cells by inhibiting cell viability through the induction of cell cycle arrest [119,122,123], necrosis [119], or apoptosis [100,101,119,120,121]. The inhibition of cell growth and proliferation may be attributed to the metabolic alterations induced by MSM at the transcriptional and/or post-translational stages. For instance, MSM has been shown to inhibit expression and DNA binding of transcription factors such as STAT3 [100,101] and STAT5b [100,101,181]; meanwhile, the p53 transcription factor is maintained by MSM [100] and does not induce apoptosis [121]. Though MSM inhibition of DNA binding by STAT3 may be an indirect effect of the phosphorylation of Jak2 [99]. Nonetheless, by inhibiting the binding of STAT3 and STAT5b to promoters, the reduced expression of oncogenic proteins such as vascular endothelial growth factor (VEGF) [99,100,101,123], heat shock protein (HSP)90α [100], and insulin-like growth factor-1 receptor (IGF-1R) [99,100,101] has been observed. The reduced expression of IGF-1R and VEGF may help prevent the development of tumors by reducing the insulin-like growth factor-1 (IGF-1)-mediated cell survival and proliferation pathways and preventing tumor-induced angiogenesis [182,183]. These metabolic alterations contribute to profound alterations at the cellular level as well.

In vitro studies with cancer cell lines suggest that MSM has the ability to stimulate phenotypic changes more closely resembling non-cancerous cells. Treatment with MSM results in the induction of contact inhibition and cell senescence [122,123,125,126], anchorage-dependent growth [122,125], reduced migration of metastatic lines [101,122,125,126], and normalized wound healing [122,125]. This could in part be attributed to the robust changes to cellular filaments, including the disassembly and indirect reassembly of microtubules [123] and reorganization of actin localization [125]. While preventing angiogenesis may prompt a state of hypoxia, MSM has also been shown to reduce levels of HIF-1α under hypoxic conditions [100,123] and prevent or improve various metastatic biomarkers in response to hypoxia [123]. In vitro MSM studies have also been supported by additional xenograft and in vivo studies confirming the results.

When cancer cells are xenotransplanted into animal models treated with MSM, tumor growth suppression has been observed [99,100,101], though two of these studies included a combination treatment of MSM and AG490 [99] or Tamoxifen [101]. Tumor tissue from mice treated exclusively with MSM exhibited reduced expression of IGF-1, STAT3, STAT5b, and VEGF without significant suppression of IGF-1R [100]. Tissues isolated from xenografted mice treated with combination treatments both displayed downregulation of STAT5b and IGF-1R signaling [99,101]. Previous studies also suggest that pre-treatment with MSM for approximately one week prior to inducing cancer in rats results in a significant reduction in the mean time to tumor onset [184,185]. Human trials with MSM as a cancer treatment have not been conducted to date; however, one study suggests that MSM use may be associated with a decreased risk of lung and colorectal cancer [186]. In vitro and in vivo results warrant further investigation of MSM as a treatment for cancer.

## 4. Safety Profile

MSM appears to be well-tolerated and safe. A number of toxicity studies have been conducted in an array of animals including rats [184,185,187,188,189], mice [190], and dogs [191,192]. In a preliminary toxicity study report, a single mortality was reported in a female rat given an oral aqueous dose of 15.4 g/kg after two days; however, a post-mortem necropsy examination showed no gross pathological alterations. Other technical reports indicate that mild skin and eye irritation have been observed when MSM is applied topically. Nonetheless, under the Food and Drug Administration (FDA) GRAS notification, MSM is considered safe at dosages under 4845.6 mg/day [25]. A summary of the toxicity studies is listed in Table 1.

### MSM and Alcohol

Much anecdotal evidence from web forums and videos exists regarding chronic MSM use and increased sensitivity to alcohol. Since other sulfur containing molecules, such as disulfiram, are used to combat alcoholism by causing adverse reactions when consuming alcohol [193], it is worth mentioning there have been no studies to date examining the effects of MSM usage on alcohol metabolism or addiction pathways. As mentioned previously, MSM readily crosses the blood brain barrier and becomes evenly distributed throughout the brain [76,77,78,79,80]; however, studies have not focused on the metabolic effects on the different neural pathways. Further studies are needed to assess the safety of MSM use with recreational alcohol use.

## 5. Conclusions

MSM is a naturally occurring organosulfur compound with broad biological effects. Human absorption and biosynthesis of this compound likely depends heavily on the co-metabolism between microbiota and host. Whether naturally produced or manufactured, MSM exhibits no biochemical differences in its ability to intermediate oxidative stress and inflammation. This micronutrient is well tolerated for arthritis and a number of other conditions related to inflammation, physical function, and performance. Emerging research suggests that MSM may one day aid in the treatment of various types of cancer [49,99,100,101,119,120,121,122,123,125,126,181,184,185,186,194] or metabolic syndromes [195].

## Figures and Tables

**Table 1:** Methylsulfonylmethane (MSM) Toxicity Data.

Species	Route	Duration	NOAEL	Reference
Acute ≤15 days				
Mice	Oral	Not stated (acute)	5 g/kg	Kocsis et al. (1975) [6]
Mice	Intraperitoneal	Not stated (acute)	5 g/kg	Kocsis et al. (1975) [6]
Mice	Oral gavage	15 days	5 g/kg	Takiyama et al. (2010) [190]
Rat	Intraperitoneal	Not stated (acute)	5 g/kg	Kocsis et al. (1975) [6]
Rat	Oral gavage	15 days	2 g/kg	Horvath et al. (2002) [187]
Subacute				
Gestating Rat	Oral gavage (14 days)	21 days	1 g/kg/day	Magnuson et al. (2007) [188]
Subchronic				
Mice	Oral	91 days	1.5 g/kg/day	Takiyama et al. (2010) [190]
Rat	Oral	90 days	1.5 g/kg/day	Horvath et al. (2002) [187]

## Abbreviations

ALDH	Aldehyde Dehydrogenase
ALF	Aggregated Locomotor Function
Bcl-2	B-cell lymphoma 2
Bcl-XL	B-cell lymphoma-extra large
BW	Body Weight
CAM	Complementary and Alternative Medicine
CAT	Catalase
CO	Carbon Monoxide
COX	Cyclooxygenase
CRP	C-Reactive Protein
DMS	Dimethyl Sulfide
DMSO	Dimethyl Sulfoxide
DMSP	Dimethylsulfoniopropionate
DNA	Deoxyribose Nucleic Acid
ECM	Extracellular Matrix
EDTA	Ethylenediaminetetraacetic acid
GCL	Glutamate-Cysteine Ligase
GCM	Glucosamine, Chondroitin Sulfate, and Methylsulfonylmethane
GPx	Glutathione Peroxidase
GRAS	Generally Recognized As Safe
GSH	Reduced Glutathione
GSSG	Oxidized Glutathione
GST	Glutathione S-Transferase
H2O2	Hydrogen Peroxide
HIF-1α	Hypoxia Inducible Factor-1α
HIV-1 Tat	Human Immunodeficiency Virus Type 1 Transactivating regulatory protein
HSP	Heat Shock Protein
IGF-1	Insulin-like Growth Factor-1
IGF-1R	Insulin-like Growth Factor-1 Receptor
IL	Interleukin
iNOS	Inducible Nitric Oxide Synthase
Jak	Janus Kinase
LD50	Lethal Dose
LPS	Lipopolysaccharide
MDA	Malondialdehyde
MPO	Myeloperoxidase
MSM	Methylsulfonylmethane
NADPH2	Reduced Nicotinamide-Adenine Dinucleotide Phosphate
NF-κB	Nuclear Factor Kappa-light-chain-enhancer of activated B cells
NHANES	National Health and Nutritional Examination Survey
NHIS	National Health Interview Surveys
NLRP3	Nucleotide-binding domain, Leucine-Rich repeat family Pyrin domain containing 3
NO	Nitric Oxide
NO3	Nitrate
NOAEL	No Observed Adverse Effect Level
Nrf2	Nuclear factor (erythroid-derived 2)-like 2
O2	Molecular Oxygen
O2-	Superoxide Radical
OA	Osteoarthritis
OH	Hydroxyl Radical
ppm	Parts per million
Prdx	Peroxiredoxin
ROS	Reactive Oxygen Species
SF36	36-Item Short Form Survey
SOD	Superoxide Dismutase
STAT	Signal Transducers and Activators of Transcription
TEAC	Trolox Equivalent Antioxidant Capacity
TNF-α	Tumor Necrosis Factor-alpha
UV	Ultraviolet
VAS	Visual Analogue Scale
VEGF	Vascular Endothelial Growth Factor
WOMAC	Western Ontario and McMaster Universities Arthritis Index
